# Healthcare professionals’ perspectives on working conditions, leadership, and safety climate: a cross-sectional study

**DOI:** 10.1186/s12913-018-3862-7

**Published:** 2019-01-21

**Authors:** Anke Wagner, Monika A. Rieger, Tanja Manser, Heidrun Sturm, Juliane Hardt, Peter Martus, Constanze Lessing, Antje Hammer, on behalf of the WorkSafeMed Consortium, E. Luntz, M. A. Rieger, H. Sturm, A. Wagner, A. Hammer, T. Manser, P. Martus, M. Holderried

**Affiliations:** 1 Institute of Occupational and Social Medicine and Health Services Research, University Hospital of Tübingen, Wilhelmstraße 27, 72074 Tübingen, Germany; 2 University of Applied Sciences and Arts Northwestern Switzerland, FHNW School of Applied Psychology, Riggenbachstrasse 16, 4600 Olten, Switzerland; 3 Institute for Clinical Epidemiology and Applied Biometry, University Hospital of Tübingen, Silcherstraße 5, 72076 Tübingen, Germany; 4 Berlin Institute of Health (BIH), Clinical Research Unit (CRU), Anna-Louisa-Karsch-Str. 2, 10178 Berlin, Germany; 5 Institute of Biometry and Clinical Epidemiology, Charité – Universitätsmedizin Berlin, Charitéplatz 1, 10117 Berlin, Germany; 6 Institute for Patient Safety, University Hospital of Bonn, Sigmund-Freud-Str. 25, 53127 Bonn, Germany; 7 indpendent consultant, Berlin, Germany

**Keywords:** Patient safety climate, Occupational safety climate, Hospital, Working conditions, Leadership, Transformational leadership

## Abstract

**Background:**

Promoting patient and occupational safety are two key challenges for hospitals. When aiming to improve these two outcomes synergistically, psychosocial working conditions, leadership by hospital management and supervisors, and perceptions of patient and occupational safety climate have to be considered. Recent studies have shown that these key topics are interrelated and form a critical foundation for promoting patient and occupational safety in hospitals. So far, these topics have mainly been studied independently from each other. The present study investigated hospital staffs’ perceptions of four different topics: (1) psychosocial working conditions, (2) leadership, (3) patient safety climate, and (4) occupational safety climate. We present results from a survey in two German university hospitals aiming to detect differences between nurses and physicians.

**Methods:**

We performed a cross-sectional study using a standardized paper-based questionnaire. The survey was conducted with nurses and physicians to assess the four topics. The instruments mainly consisted of scales of the German version of the COPSOQ (Copenhagen Psychosocial Questionnaire), one scale of the Copenhagen Burnout Inventory (CBI), scales to assess leadership and transformational leadership, scales to assess patient safety climate using the Hospital Survey on Patient Safety Culture (HSPSC), and analogous items to assess occupational safety climate.

**Results:**

A total of 995 completed questionnaires out of 2512 distributed questionnaires were returned anonymously. The overall response rate was 39.6%. The sample consisted of 381 physicians and 567 nurses. We found various differences with regard to the four topics. In most of the COPSOQ and the HSPSC-scales, physicians rated psychosocial working conditions and patient safety climate more positively than nurses. With regard to occupational safety, nurses indicated higher occupational risks than physicians.

**Conclusions:**

The WorkSafeMed study combined the assessment of the four topics psychosocial working conditions, leadership, patient safety climate, and occupational safety climate in hospitals. Looking at the four topics provides an overview of where improvements in hospitals may be needed for nurses and physicians. Based on these results, improvements in working conditions, patient safety climate, and occupational safety climate are required for health care professionals in German university hospitals – especially for nurses.

**Electronic supplementary material:**

The online version of this article (10.1186/s12913-018-3862-7) contains supplementary material, which is available to authorized users.

## Background

Promoting patient and occupational safety are two key challenges for hospitals. To effectively manage these challenges, healthcare organizations are recommended to develop a culture of safety [1]. An organizations safety culture refers to “the product of individual and group values, attitudes, perceptions, competencies, and patterns of behaviour that determine the commitment to, and the style and proficiency of an organization’s health and safety management. Organizations with a positive safety culture are characterized by communications founded on mutual trust, by shared perceptions of the importance of safety and by confidence in the efficacy of preventive measures” [1]. In summary, an organization’s safety culture reflects how safety is viewed and treated in organizations [2], guiding employees and hospital managers in fulfilling their tasks and in dealing with safety issues [3]. Patient safety can be therefore defined as “the avoidance, prevention and amelioration of adverse outcomes or injuries stemming from the process of healthcare” [4]. Occupational safety and occupational safety climate relates to workplace health and safety and deals with workgroup members’ shared perceptions of policy, procedures, and practice in relation to occupational health and safety in the organization [5].

Given the dynamic nature of modern hospitals, healthcare professionals are confronted with major changes in psychosocial working conditions characterized by skills shortage or imbalance, increasing workload and task complexity [6–10]. In addition, demographic changes are making hospital-based patient care increasingly demanding, as chronic diseases and multimorbidity are becoming more predominant [6, 10–12]. To support adaptation to the dynamically evolving nature of work in hospitals, leadership by hospital management and direct supervisors takes on a central role [13, 14]. A transformational leadership style has been shown to contribute particularly well to high performance in the face of organizational change [15–21]. Especially in safety-critical working environments, transformational leadership is positively associated with employees’ safety performance and behaviour [21, 22]. It has been shown to increase employees’ level of awareness regarding organizational learning processes and the importance of accomplishments and to support their commitment towards common missions [23, 24].

In recent years, several studies on working conditions [6, 25–32], on (transformational) leadership [17–20, 33], and on patient safety climate in hospitals [34–36] have been published. While there are a great number of studies investigating the association between working conditions and safety climate in hospitals [37–41], there are only few studies focusing explicitly on occupational safety climate in hospitals [42, 43], or investigating the association between patient and occupational safety climate [44–46]. As a common result, studies have shown that these four key topics - (1) psychosocial working conditions, (2) leadership, (3) patient safety climate, and (4) occupational safety climate - are interrelated and form a critical foundation for promoting patient and occupational safety in hospitals. However, relevant studies mentioned above clearly show that these topics have mainly been studied independently of each other and in most cases solely focus on one professional group, either nurses or physicians.

Previous studies showed that physicians and nurses perceptions on psychosocial working conditions and safety culture vary, although they work in the same setting [36, 47]. Recently conducted studies also identified close relationships between working environments for hospital staff and safety culture [48, 49]. Thus, it can be assumed that improving working conditions for healthcare professionals also leads to improved safety culture.

Moreover, when aiming to improve both patient and occupational safety in hospitals, psychosocial working conditions, leadership by hospital management and supervisors, and perceptions of occupational and patient safety climate have to be considered. Consequently, studies aiming to assess and potentially improve occupational as well as patient safety climate should take into account the views of nurses and physicians. Likewise, it is important to assess and evaluate perceptions and attitudes of the closely cooperating frontline healthcare workers to these four topics in order to develop comprehensive improvement measures for patient and occupational safety culture in hospitals.

The present study investigated hospital staffs’ perception of these four topics for the first time from the perspectives of both nurses and physicians. We present descriptive findings on the current state in two German university hospitals and investigate perceptions and attitudes of nurses and physicians related to these four topics aiming to detect possible differences.

## Methods

### Study design and questionnaire

Between 2014 and 2017 we conducted a cross-sectional, multicenter, mixed-methods project *Working conditions, safety culture and patient safety in hospitals – what predicts the safety of the medication process* (WorkSafeMed). Part of the WorkSafeMed project was a staff survey using a standardized paper-based questionnaire. An overview of all scales and items used in this paper is provided in Table 1.

**Table 1:** Overview of the scales and items presented in this paper

Assessment	Psychosocial working conditions	Leadership	Patient safety climate	Occupational safety climate
Instruments	Copenhagen Psychosocial Questionnaire (COPSOQ) and adaptation of Copenhagen Burnout Inventory (CBI)	Transformational Leadership Inventory (TLI short) and Copenhagen Psychosocial Questionnaire (COPSOQ)	Hospital Survey on Patient Safety Culture (HSPSC-D) and Twins Patient Safety	TWINS Occupational Safety and self constructed indices
Overall number of scales and items	*17 scales*	*2 scales*	*13 scales and 9 single items*	*1 scale, 3 indices, 7 single items*
Scales, indices and items	*COPSOQ* – Quantitative demands (scale, 4 items) – Emotional demands (scale, 3 items) – Work-privacy-conflict (scale, 5 items) – Influence at work (scale, 4 items) – Degree of freedom at work (scale, 4 items) – Possibilities for development (scale, 4 items) – Meaning of work (scale, 3 items) – Workplace commitment (scale, 4 items) – Predictability (scale, 2 items) – Role clarity (scale, 2 items) – Role conflicts (scale, 4 items) – Social relations (scale, 2 items) – Feedback (scale, 2 items) – Social support (scale, 4 items) – Sense of community (scale, 3 items) *Outcome scale - COPSOQ* – Job satisfaction (scale, 7 items) *Outcome scale - CBI (adapted client-related burnout)* – Patient-related burnout (scale, 6 items)	*TLI short* – Transformational leadership (scale, 6 items) *COPSOQ* – Quality of leadership (scale, 4 items)	*HSPSC-D* – Teamwork within units (scale, 4 items) – Staffing (scale, 4 items) – Organizational learning (scale, 3 items) – Nonpunitive response to error (scale, 3 items) – Supervisor/ manager expectations (scale, 4 items) – Feedback & communication about error (scale, 3 items) – Communication openness (scale, 3 items) – Management support for patient safety (scale, 3 items) – Teamwork across units (scale, 4 items) – Handoffs & transitions (scale, 4 items) *Outcome scales - HSPSC-D* – Frequency of event reported (scale, 3 items) – Overall perceptions of patient safety (scale, 4 items) – Patient safety grade (single item) – Safety grade in the medication process (single item) *TWINS Patient Safety* – Supervisor support for patient safety (scale, 3 items) – My direct supervisor openly addresses problems concerning patient safety in our hospital (single item) – My direct supervisor focuses more on patient safety than a year ago (single item) – It is important to my direct supervisor that our hospital pays great attention to patient safety (single item) – Hospital management openly addresses problems concerning patient safety in our hospital (single item) – Hospital management focuses more on patient safety than a year ago (single item) – It is important to the hospital management that our hospital pays great attention to patient safety (single item) – Do you have an individual influence on how well patient safety is implemented at the workplace (single item)	*TWINS Occupational Safety* – Supervisor support for occupational safety (scale, 3 items) – My direct supervisor openly addresses problems concerning occupational safety in our hospital (single item) – My direct supervisor focuses more on occupational safety than a year ago (single item) – It is important to my direct supervisor that our hospital pays great attention to occupational safety (single item) – Hospital management openly addresses problems concerning occupational safety in our hospital (single item) – Hospital management focuses more on occupational safety than a year ago (single item) – It is important to the hospital management that our hospital pays great attention to occupational safety (single item) – Do you have an individual influence on how well occupational safety is implemented at the workplace (single item) *Outcome scales – self constructed indices* – Subjective assessment of specific protective measures (behaviour & regulations) related to infectious diseases (index, 7 items) – Subjective assessment of occupational safety measures initiated by the employer, related to own safety (index, 6 items) – Personal perception of the frequency of occupational risks (index, 4 items)

The questionnaire used common and validated instruments to measure four study topics:

1. *Psychosocial working conditions:* To measure staffs’ perceptions of psychosocial working conditions and the according strain (job satisfaction and burnout), we used 16 scales, each with a number of items ranging between three to seven, from the German version of the Copenhagen Psychosocial Questionnaire (COPSOQ) [50–52]. The COPSOQ comprises concepts from several traditional theories of psychosocial working conditions, e. g. the job demand-control model by Karasek [53] with the established scales “influence at work” and “degree of freedom at work”. Single items were rated on a 4-point and 5-point Likert scale. We also adapted one scale from the Copenhagen Burnout Inventory (client-related burnout) to measure patient-related burnout [54]. Before calculating scale scores for each dimension and in accordance with the recommended COPSOQ transformation [52], scales were transformed into scores ranging from 0 (minimum value, “do not agree at all”) to 100 points (maximum value, “fully agree”). Negatively worded items were not recoded in the process of documentation. However, depending on the wording of items within each scale, maximum values can be positive (high = positive) or negative (high = negative). For example: A high value for “influence at work” is considered positive while a high value for “quantitative demands” is considered negative.
2. *Leadership:* To measure leadership, and especially transformational leadership, we focused on the leadership quality scale from the COPSOQ-questionnaire [50, 51] and the short scale on Transformational Leadership (TLI-short) [19]. The latter is a shortened measure derived from a German adaption of the Transformational Leadership Inventory (TLI) [55, 56]. Each of the six TLI-short items matched one of the six transformational behaviours in the original inventory by reflecting the item with the highest factor loading within the German TLI [19]. The items of the TLI short scale were answered on a 5-point Likert scale of frequency (from “1 = never” to “5 = always”), where high values imply a high perception on transformational leadership. The items on the leadership quality scale from the COPSOQ-questionnaire were rated on a 5-point Likert scale. As above, answer scales were transformed into scale scores ranging from 0 (minimum value, “do not agree at all”) to 100 points (maximum value, “fully agree”). Due to the wording of the scale items, maximum values are positive (high = positive).
3. *Patient safety climate*: The multi-dimensional construct of safety culture is usually quantitatively measured by safety climate, which can be defined as the shared perceptions of employees about safety-relevant aspects of their work environment [57, 58]. To assess patient safety climate, we used the German version of the *Hospital Survey on Patient Safety Culture* (HSPSC-D) [59]. The instrument used in this study consisted of 43 items, measuring ten patient safety culture scales, two outcome scales; one single-item outcome on *patient safety grade*, and one single-item outcome on the *overall safety grade in the medication process*. Scale-items were rated on a 5-point Likert scale, either of agreement (from “1 = strongly disagree” to “5 = strongly agree”) or frequency (from “1 = never” to “5 = always”). Scale scores were calculated after reverse coding of negatively worded items. High values on scales imply a high perception on patient safety climate. The two single-item outcomes were answered on a 5-point Likert frequency scale ranging from “1 = excellent” to “5 = failing”, where high values imply a rather low perception of these two outcomes. Based on findings from a former study [60], self-developed items of the HSPSC-D measuring aspects of supervisor and management support regarding patient safety was used to capture the interaction of supervisors and management from the participants´ perspective. Hereby, the original HSPSC-D scale “management support for patient safety” was worded analogously to cover the specific aspects with regard to the supervisor’s support (new scale “supervisor’s support for patient safety” – the original scale was omitted) and a set of items covering both the role of supervisors and the management were developed. In a second step, this set of items was also verbalized with regard to occupational safety climate (see below) and both sets of items were named as “twins” (TWINS Patient Safety). Each item of the TWINS Patient Safety was rated on a 5-point Likert scale of agreement (from “1 = strongly disagree” to “5 = strongly agree”) or frequency (from “1 = never” to “5 = always”). Maximum values are positive except for one item (*Individual influence on patient safety at the workplace*), where high values imply a rather low perception of one’s own influence.
4. *Occupational safety climate:* As described above for the patient safety climate, we employed an identic item set to capture aspects of supervisor and management support regarding occupational safety as important aspect of the occupational safety climate (TWINS Occupational Safety). Each item here was rated on a 5-point Likert scale of agreement (from “1 = strongly disagree” to “5 = strongly agree”) or frequency (from “1 = never” to “5 = always”). Maximum values are positive except for one item (*Individual influence on occupational safety at the workplace*), where high values imply a rather low perception of one’s own influence. To assess occupational safety climate outcomes, we used three self-constructed indices (good Cronbach’s alpha from .76 to .82), which measure perceived occupational safety: (1) subjective assessment of specific protective measures (behaviour & regulations) related to work-related infectious diseases (e.g. protective gloves), (2) subjective assessment of occupational safety measures initiated by the employer, related to own safety (e.g. regulations on how to act in the case of fire or other emergency) and (3) personal perception of the frequency of occupational risks (e.g. do you feel exposed to risks of infection?). Items were answered on a 5-point Likert scale of agreement (from “1 = strongly agree” to “5 = strongly disagree”) or frequency (from “1 = never” to “5 = always”). Low values on scales and single items imply a high perception of occupational safety climate.

Prior to data collection, the final survey underwent a pre-test with 4 physicians and 8 nurses using cognitive think aloud interviews.

### Setting and sample

We conducted the staff survey with healthcare professionals at two German university hospitals. Hospital selection was based on a convenient sample to have an appropriate sample size large enough to perform multivariate analyses and keep organizational characteristics as comparable as possible. We included all inpatient units, which treat at least 500 patients per year and excluded intensive care and psychiatric units.

### Data collection

Prior to data collection, the consent of the executive board of directors, the workers council, and the medical directors of the clinics/departments participating in the study was obtained in both university clinics. After a hospital-wide information by the executive medical directors of the two participating university clinics, the study was presented in department meetings of physicians or during regular team meetings of nurses in the units. The questionnaire then was distributed to a total of 2512 physicians and nurses (including nursing aids and nurses in vocational training). In total, we collected data from 37 departments including 73 units. The data collection took place between April 2015 and July 2015. After approximately two to four weeks, at least one written and, if necessary, oral reminder was carried out on the level of departments (physicians) or units (nurses).

### Statistical analysis

Prior to data analyses, we imputed missing values in the survey data (excluding sociodemographic items). For this, scale items from the four different topics (psychosocial working conditions, leadership, patient safety climate, occupational safety climate) were grouped into four separate imputation groups. Within each imputation group, respondents with missing values of > 30% for scale items were excluded because of the limited data quality (Respondents with missing values: Imputation group 1 (psychological working conditions): *n* = 4 (0,4%), imputation group 2 (leadership): *n* = 42 (4,2%), imputation group 3 (patient safety climate): *n* = 21 (2,1%), imputation group 4 (occupational safety climate): *n* = 22 (2,2%)). Then data for each group were imputed with NORM 2.03 software using the Expectation-Maximization-algorithm [61, 62]. After the necessary reverse coding of negatively worded items, mean scale values were computed for all scales of the four topics. Descriptive analyses included mean values and standard deviations (mean ± SD) of continuous variables and scale-scores, and absolute and percentage frequencies of categorical variables. T-tests for independent samples were used to determine differences in mean values between nurses and physicians. *P*-values ≤.05 were considered statistically significant. As this is an explorative study, significance testing was conducted to discover tendencies and not for confirmatory purposes, thus no adjustment for multiple testing was applied. We calculated and categorized the effect size according to Cohen’s suggestions: mean/SD < .30 = small effect/difference, <.50 = medium effect/difference and ≥ .50 = large effect/difference [63]. Data were analysed using IBM Statistics SPSS (Version 23) for Windows. We found some statistically significant differences between the two hospitals: Overall, psychosocial working conditions at the first hospital were indicated more positively than at the second hospital. Patient safety culture also received more positive ratings at this hospital. However, the differences in most of the scales represent only small effects (for more information see Additional file 1) and are not relevant for answering our research question. Therefore, all descriptive results are presented for both hospitals together.

### Ethics and confidentially issues

Ethics approval was obtained from the ethical committees at the two participating university hospitals. Informed consent was sought from participants, who were informed that the study was voluntary and that they could withdraw at any time. The data were analysed anonymously.

## Results

### Response rate and sample characteristics

A total of 995 out of 2512 distributed questionnaires were completed and returned. Thus, the overall response rate was 39.6%. The sample consisted of 381 physicians and 567 nurses (including nursing aids and nurses in vocational training). The response rates were 39.4% for nurses and 35.5% for physicians. In addition, 47 persons participated who either belonged to another professional group (19 persons) or gave no information on their professional status (28 persons). The characteristics of the sample are summarized in Table 2.

**Table 2:** Demographic characteristics of the study respondents

Characteristic of the study respondents	*N*	%
Profession		
Nurse	567	57.0%
Physician	381	38.3%
Others	19	1.9%
Missing	28	2.8%
Gender		
Male	291	29.2%
Female	656	65.9%
Missing	48	4.8%
Supervisor function		
Yes	195	19.6%
No	759	76.3%
Missing	41	4.1%
	Mean (SD)	Range in years
Age	37,7 (10,7)	19 to 65
Average work experience	13,5 (10,9)	0 to 44
Average work experience in the hospital	10,7 (9,5)	0 to 43
Average work experience in the current department	8,5 (8,2)	0 to 40

Descriptive results including differences for nurses and physicians in scale scores and items are presented on Table 3.

**Table 3:** Descriptive statistics, results of the student’s t test and effect size comparing answers by nurses and physicians

Psychosocial working conditions	Interpretation (0 = minimum value, 100 = maximum value)	Mean (SD) (nurses = 564)	Mean (SD) (physicians = 380)	(df) t-value^1^	d_Cohen_
Copenhagen Psychosocial Questionnaire (COPSOQ)					
Quantitative demands	high = negative	66.5 (13.5)	71.9 (13.9)	(942) -5.974*	0.40
Emotional demands	high = negative	64.4 (18.3)	64.6 (16.5)	(942) -.202	0.01
Work-privacy-conflict	high = negative	61.3 (24.4)	68.7 (25.1)	(942) -4.497*	0.30
Influence at work	high = positive	36.3 (17.3)	38.8 (20.8)	(710) -2.006*	0.13
Degree of freedom at work	high = positive	36.0 (15.9)	46.2 (20.0)	(687) -8.373*	0.58
Possibilities for development	high = positive	71.6 (15.7)	79.6 (14.2)	(942) -8.032*	0.53
Meaning of work	high = positive	77.7 (16.6)	82.9 (16.1)	(942) -4.753*	0.32
Workplace commitment	high = positive	48.4 (18.8)	61.3 (19.2)	(942) -10.220*	0.68
Predictability	high = positive	53.3 (16.4)	52.5 (19.3)	(720) 0.710	−0.05
Role clarity	high = positive	73.5 (14.5)	72.5 (16.5)	(740) 1.027	−0.07
Role conflicts	high = negative	50.6 (17.2)	45.1 (18.4)	(942) 4.611*	−0.31
Feedback	high = positive	41.9 (21.0)	41.0 (21.5)	(942) 0.632	−0.04
Social support	high = positive	66.7 (17.0)	64.2 (17.0)	(942) 2.169*	−0.15
Social relations	high = positive	45.0 (17.0)	51.5 (15.1)	(874) -6.194*	0.40
Sense of community	high = positive	77.8 (15.2)	76.7 (15.1)	(942) 1.096	−0.07
Outcome scale – Copenhagen Psychosocial Questionnaire (COPSOQ)					
Job satisfaction	high = positive	67.5 (10.2)	73.4 (12.0)	(942) -8.135*	0.54
Outcome scale – Copenhagen Burnout Inventory (CBI, adapted client-related burnout)					
Patient related burnout	high = negative	36.5 (17.6)	28.0 (16.5)	(942) 7.464*	−0.50
Leadership	Interpretation (0/1 = minimum value, 100/5 = maximum value)	Mean (SD) (nurses = 543)	Mean (SD) (physicians = 369)	(df) t-value^1^	d_Cohen_
Transformational Leadership Inventory (TLI short)					
Transformational leadership	5 = positive	3.1 (0.8)	3.2 (0.8)	(910) -1.605	0.13
Copenhagen Psychosocial Questionnaire (COPSOQ)					
Quality of leadership	high = positive	53.8 (22.7)	49.2 (22.9)	(910) 3.031*	−0.20
Patient safety climate	Interpretation (1 = minimum value, 5 = maximum value)	Mean (SD) (nurses = 558)	Mean (SD) (physicians = 373)	(df) t-value^1^	d_Cohen_
Hospital Survey on Patient Safety Culture (HSPSC-D)					
Staffing	5 = positive	2.4 (0.8)	2.8 (0.8)	(929) -7.721*	0.50
Organizational learning	5 = positive	3.0 (0.7)	3.1 (0.7)	(762) -1.366	0.14
Communication openness	5 = positive	3.7 (0.6)	3.4 (0.7)	(758) 6.010*	−0.47
Feedback & communication about error	5 = positive	3.4 (0.8)	3.3 (0.9)	(929) 1.519	−0.12
Nonpunitive response to error	5 = positive	3.3 (0.8)	3.5 (0.8)	(929) -3.746*	0.25
Teamwork within units	5 = positive	3.3 (0.6)	3.4 (0.6)	(929) 1.326	0.17
Teamwork across units	5 = positive	3.0 (0.6)	3.1 (0.7)	(698) -3.316*	0.16
Handoffs & transitions	5 = positive	3.2 (0.6)	2.9 (0.7)	(713) 5.702*	−0.47
Supervisor/manager expectations	5 = positive	3.4 (0.7)	3.3 (0.7)	(929) 1.020	−0.14
Management support for patient safety	5 = positive	2.6 (0.8)	3.0 (0.8)	(929) -5.797*	0.50
Outcome scales – Hospital Survey on Patient Safety Culture (HSPSC-D)					
Frequency of event reported	5 = positive	3.0 (1.1)	2.9 (0.9)	(874) 1.053	−0.10
Overall perceptions of patient safety	5 = positive	2.9 (0.7)	3.3 (0.8)	(929) -7.782*	0.54
Patient safety grade	1 = positive	2.9 (0.8)	2.6 (0.7)	(929) 7.456*	−0.39
Safety grade in the medication process	1 = positive	3.0 (0.8)	2.8 (0.7)	(831) 5.065*	−0.26
Patient safety climate	Interpretation (1 = minimum value, 5 = maximum value)	Mean (SD) (nurses = 543)	Mean (SD) (physicians = 369)	(df) t-value^1^	d_Cohen_
TWINS Patient Safety					
Supervisor support for patient safety	5 = positive	3.4 (0.8)	3.5 (0.7)	(910) -1.996*	0.13
My direct supervisor openly addresses problems concerning patient safety in our hospital	5 = positive	3.3 (0.9)	3.3 (1.0)	(729) -0.865	0.00
My direct supervisor focuses more on patient safety than a year ago	5 = positive	2.8 (0.9)	2.8 (1.0)	(735) -0.27	0.00
It is important to my direct supervisor that our hospital pays great attention to patient safety	5 = positive	3.4 (0.9)	3.5 (0.9)	(910) -1.509	0.11
Hospital management openly addresses problems concerning patient safety in our hospital	5 = positive	2.8 (0.8)	3.0 (0.9)	(910) -4.188*	0.36
Hospital management focuses more on patient safety than a year ago	5 = positive	2.7 (0.9)	2.8 (0.9)	(910) -2.758*	0.12
It is important to the Hospital management that our hospital pays great attention to patient safety	5 = positive	3.0 (1.0)	3.2 (1.0)	(784) -3.698*	0.20
Do you have an individual influence on how well patient safety is implemented at the workplace	1 = positive	3.2 (0.9)	2.9 (1.0)	(910) 4.558*	−0.32
Occupational safety climate	Interpretation (1 = minimum value, 5 = maximum value)	Mean (SD) (nurses = 543)	Mean (SD) (physicians = 369)	(df) t-value^1^	d_Cohen_
TWINS Occupational Safety					
Supervisor support for occupational safety	5 = positive	3.5 (0.8)	3.4 (0.8)	(910) 1.050	−0.13
My direct supervisor openly addresses problems concerning occupational safety in our hospital	5 = positive	3.3 (0.9)	3.2 (0.9)	(910) 0.869	0.00
My direct supervisor focuses more on occupational safety than a year ago	5 = positive	2.8 (0.9)	2.7 (0.9)	(910) 0.628	−0.11
It is important to my direct supervisor that our hospital pays great attention to occupational safety	5 = positive	3.3 (0.9)	3.2 (1.0)	(910) 2.299*	−0.11
Hospital management openly addresses problems concerning occupational safety in our hospital	5 = positive	2.9 (0.9)	3.1 (0.9)	(910) -3.337*	0.22
Hospital management focuses more on occupational safety than a year ago	5 = positive	2.7 (0.9)	2.8 (0.9)	(910) -1.936	0.11
It is important to the Hospital management that our hospital pays great attention to occupational safety	5 = positive	2.9 (0.9)	3.1 (1.0)	(766) -2.720*	0.21
Do you have an individual influence on how well occupational safety is implemented at the workplace	1 = positive	3.3 (0.9)	3.3 (1.0)	(910) .893	0.00
Occupational safety climate	Interpretation (1 = minimum value, 5 = maximum value)	Mean (SD) (nurses = 560)	Mean (SD) (physicians = 372)	(df) t-value^1^	d_Cohen_
Outcome scales – self constructed indices					
Subjective assessment of specific protective measures (behaviour & regulations) related to infectious diseases	1 = positive	1.8 (0.6)	1.8 (0.6)	(930) -1.132	0.00
Subjective assessment of occupational safety measures initiated by the employer, related to own safety	1 = positive	1.7 (0.6)	2.0 (0.6)	(930) -8.328*	0.50
Personal perception of the frequency of occupational risks	5 = positive	3.2 (0.8)	3.5 (0.7)	(853) -5.608*	0.39

Notes: ^1^*p*-value* ≤.05

### Psychosocial working conditions

#### Psychosocial working conditions

When analysing demands, we found high values for both professional groups. *Quantitative demands* were rated higher than *emotional demands*. The mean score of the *work-privacy-conflict* scale was also high in both professional groups. When comparing the two professional groups, we found that physicians experienced significantly greater *quantitative demands* (71.9 ± 13.9) than nurses (66.5 ± 13.5). However, there were no significant differences in *emotional demands*. Furthermore, although high in both professional groups, physicians reported a significantly greater *work-privacy-conflict* (68.7 ± 25.1) than nurses (61.3 ± 24.4). Both differences represented medium effects (quantitative demands: d = .40; work-privacy-conflict: d = .30).

There were medium value ranges given for *influence at work*, *degree of freedom at work,* and *workplace commitment*, while high (positive) value ranges were reported for *possibilities for development* and *meaning of work.* All in all, physicians made more positive indications in this domain than the nurses (see Table 3). Differences of the three scales *degree of freedom at work* (d = .58), *possibilities for development* (d = .53), and *workplace commitment* (d = .68) presented a large effect, while the other two scales (*influence at work*: d = .13 and *meaning of work*: d = .32) represented small to medium effects.

The results for interpersonal relations showed medium or high value ranges. Overall, we found fewer differences between physicians and nurses. There were no statistically significant differences between the two professional groups in the four scales *predictability*, *role clarity*, *feedback,* and *sense of community*. We identified significant differences with small or medium effects in three scales (*social support*: d = −.15, *role conflicts*: d = −.31, and *social relations*: d = .40). Nurses experienced more role conflicts (50.6 ± 17.2) than did the physicians (45.1 ± 18.4) in our sample. Concurrently, the results also indicate that nurses experienced more *social support* (66.7 ± 17.0) compared to physicians (64.2 ± 17.0). Physicians rated items on the scale *social relations* more positively (51.5 ± 15.1) than the nurses (45.0 ± 17.0).

#### Outcome scales

The average mean on the scale *job satisfaction* was high in both professional groups, while the results of the scale *patient-related burnout* were low. However, physicians had significantly higher values for *job satisfaction* (73.4 ± 12.0) than the nurses (67.5 ± 10.2). Similarly, physicians reported significantly fewer symptoms for *patient-related burnout* (28.0 ± 16.5) compared to nurses (36.5 ± 17.6). The differences between the professional groups with regard to *job satisfaction* and *patient-related burnout* represented a large effect size (*job satisfaction:* d = .54 and *patient-related burnout:* d = −.50).

### Leadership

Values for employees’ views on transformational leadership were relatively high for both physicians (3.2 ± 0.8) and nurses (3.1 ± 0.8). There was no significant difference in rating transformational leadership. Nurses rated the quality of leadership more positively (53.8 ± 22.7) than physicians (49.2 ± 22.9). This difference was significant but represented a small effect size (d = −.20).

### Patient safety climate

#### Patient safety climate

We observed statistically significant differences between nurses and physicians in six out of ten patient safety culture scales. Physicians gave significantly higher ratings for the four scales *staffing* (2.8 ± 0.8), *nonpunitive response to error* (3.5 ± 0.8) *management support for patient safety* (3.0 ± 0.8), and *teamwork across units* (3.1 ± 0.7) than nurses (*staffing*: 2.4 ± 0.8; *nonpunitive response to error*: 3.3 ± 0.8; *management support for patient safety*: 2.6 ± 0.8; *teamwork across units*: 3.0 ± 0.6). By contrast, nurses gave significantly higher ratings for the two scales *communication openness* (3.7 ± 0.6) and *handoffs and transitions* (3.2 ± 0.6) than the physicians (*communication openness*: 3.4 ± 0.7; *handoffs and transitions*: 2.9 ± 0.7). All of these differences represented a medium to rather large effect size, with exception of the scales *teamwork across units* and *nonpunitive response to error*. We observed no significant differences between the two professional groups in the remaining four scales (*teamwork within the units, organizational learning, supervisor/manager expectations,* and *feedback and communication about error*).

#### TWINS patient safety

We also identified significant differences for the twin items regarding patient safety climate. Physicians rated the three scales focusing on management and the scale addressing individual influence on patient safety at the workplace more positively than nurses. These differences represented a small to medium effect size. We found no significant differences between the two professional groups in the other four scales.

#### Outcome scales and items

The single items *patient safety grade* and *safety grade in the medication process* were rated significantly less safe by the nurses (*patient safety grade*: 2.9 ± 0.8; *safety grade in the medication process*: 3.0 ± 0.8) than by physicians (*patient safety grade*: 2.6 ± 0.7; *safety grade in the medication process*: 2.8 ± 0.7). In addition, physicians rated the *overall perceptions of patient safety* as significantly safer (3.3 ± 0.8) than the nurses (2.9 ± 0.7). These differences represented a medium to large effect. We found no significant difference in frequency of reported events.

### Occupational safety climate

#### TWINS occupational safety

We identified significant differences for the twin items covering occupational safety climate. Physicians rated two of the three scales focusing on management more positively than the nurses (see Table 3). These differences represented a small effect. We found no significant differences between the two professional groups in the other six scales. Overall, the two professional groups rated *individual influence on occupational safety* less positively than *individual influence on patient safety*.

#### Outcome scales – Perceived occupational safety climate

Two significant differences between the two professional groups were found in the outcome scales. Nurses rated *occupational safety measures initiated by the employer* more positively than physicians. This difference represented a large effect (d = .50). They also indicated higher occupational risks (3.2 ± 0.8) than physicians (3.5 ± 0.7). This difference was significant and it also represents a medium effect (d = .39). Both professionals groups also stated that specific protective measures related to infectious diseases were important.

## Discussion

This paper analysed data from a staff survey conducted at two German university hospitals. The applied standardized questionnaire was used to assess psychosocial working conditions, job satisfaction, patient safety climate, and occupational safety climate. We report results of descriptive and inferential statistics aiming to detect differences between the two professional groups.

### Psychosocial working conditions

Overall, there are few studies which use the COPSOQ instrument to jointly question physicians and nursing staff on their psychosocial working conditions and to compare the results. Ilic et al. questioned nurses and physicians on their working conditions and found some differences between the two professional groups [64]. The physicians in the study indicated, for example, higher demands, more influence at work, and more possibilities for development than the nurses. However, the study population of Ilic et al. consisted of nurses and physicians in emergency medicine.

Our study found significant differences between the two professional groups in 12 out of 17 scales. Nine scales (*influence at work, degree of freedom at work, possibilities for development, meaning of work, workplace commitment, role conflicts, social relations, job satisfaction*, and the additional scale *patient-related burnout*) were significantly more positively assessed by physicians than the nursing staff. This may be due to the fact that some of the differences also lie in the work characteristics of the two occupational groups. A physician usually has more influence at work than a nurse. Nursing staff assessed a total of three scales addressing the concept of psychosocial working conditions (*quantitative demands, work-privacy conflict*, and *social support*) significantly more positively than physicians. The results imply that, on the whole, the surveyed physicians in our study evaluated their psychosocial working conditions more positively than nursing staff. That nurses critically assess their working conditions was also demonstrated in other studies. For example in the RN4Cast study, in nine out of 12 European countries more than half of the surveyed nurses reported that the work environment at their hospital was poor or fair, as opposed to good or excellent [65]. Germany was one of the countries where working conditions of nurses were criticized [65]. This is not surprising, considering how, in recent years, the nursing profession in Germany has been particularly characterized by skills shortages and a shortage of freshly graduated nurses [6–10]. Due to demographic changes and an increase in patients with chronic diseases and multimorbidities, the care demands on nurses have also been steadily increasing [6, 10–12]. A previously conducted study comparing nurse emigration in Germany to nurse emigration in other countries identified poor working conditions as one of the main causes, and suggested Germany should invest in better working conditions for nurses [66]. Based on our results, measures to improve psychosocial working conditions for healthcare professionals in hospitals are necessary – with a special emphasis on improvements for nurses. The following implications to improve psychosocial working conditions for healthcare professionals in university hospitals seem to be necessary: reduction of high quantitative demands and role conflicts, and improvement of the perceived work-privacy conflict. Also, existing resources, such as social support, possibilities for development, meaning of work, sense of community should be further supported. Especially for nurses, workplace commitment and the degree of freedom at work should be improved.

### Leadership

In the concept leadership, the values for *transformational leadership* and *leadership quality* were situated in the moderate range and comparable for recently conducted studies in hospital settings [17, 19, 30, 67]. On the whole, nurses assessed the quality of leadership more positively than physicians. We presume, therefore, that the nurses were more satisfied with their direct supervisors than the physicians. The different assessment may also be due to the fact, that different work structures of physicians and nurses affect how leadership is perceived [68]. Nurses work with a direct supervisor on the ward while physicians may work in several units [68] and thus may experience less direct support by their supervisors than nurses. In Germany, it is common practice for nurses’ direct supervisors to work on site and act as a contact person. Physicians in Germany do not always have contact with their direct supervisors and may therefore assess the quality of leadership more critically. There were no significant differences in how transformational leadership was rated. However, we found only small differences between the two professional groups for both scales. According to the results, the quality of leadership can be further enhanced.

### Patient safety climate

We found significant differences between the occupational groups for patient safety climate in nine out of 14 scales. Similar to the assessment of the psychosocial working conditions, patient safety climate was also assessed more positively by physicians than by nursing staff. Seven scales (*staffing, nonpunitive response to error, teamwork across units, management support for patient safety, overall perceptions of patient safety, patient safety grade*, and *safety grade in the medication process*) were rated more positively by physicians than nurses. In contrast, nursing staff rated the scales *communication openness* and *handoffs and transitions* more positively than physicians. Our results correspond to other studies which questioned both physicians and nursing staff about patient safety and reported apparent differences between occupational groups [69–71], and that patient safety climate scales were also rated more positively by physicians than by nursing staff [71, 72]. Singer and colleagues considered whether nurses perceive safety deficiencies in organizational structures more often than physicians [71]. Another explanation is that the perceived worsening of working conditions for nurses also affects the perception of patient safety. A recently conducted study identified relationships between working environments for nurses (nurse staffing) and patient safety (increased survival of in-hospital cardiac arrest patients) [48]. The RN4Cast study investigated associations between nurse staffing, education and hospital mortality in nine European countries [49]. As a major result an increase in nurses’ workload by one patient increased the likelihood of an inpatient dying within 30 days of admission [49]. We therefore assume that improving working conditions and staffing also leads to improved patient safety.

There were also significant differences between the occupational groups for the TWINS Patient Safety, especially for the items regarding support from management. With a specific focus on patient safety, physicians rated the items regarding *hospital management* and *supervisor support for patient safety* significantly more positively than the nurses. This result is consistent with results of other studies [65, 69, 72]. In another study, nurses reported that management does not listen and answer to employee concerns, so nurses indicated that patient safety is not a management priority [65]. In the current study, nurses assessed management support for patient safety much worse compared to physicians [69, 72]. A possible explanation for this finding may be that nurses in our sample have little contact with hospital management and may therefore assume that managing staff is not interested in patient safety issues in their unit. Other authors assume that physicians work more closely with management and therefore perceive more support [72]. But in another study, physicians also indicated that the higher management does not listen and can jeopardize patient safety [73]. Overall, in our study it seems necessary for hospital management to become more visible especially to nurses and for communication between hospital management and nursing staff to be improved.

### Occupational safety climate

The TWINS Occupational Safety found significant differences between the occupational groups with a small effect in three scales. Here, similar to patient safety climate, physicians assessed the individual items related to management more positively than the nurses. On the other hand, the nurses rated the item regarding the direct supervisor more positively than the physicians. In this case, it also seems plausible that nurses are more critical of the hospital management than of their direct supervisors, since they usually have little contact with the managing staff. This result is in line with another study. Among other things, Eklöf et al. confirmed the critical evaluation of hospital management in terms of safety. A direct implication here is also to improve communication between hospital management and nursing staff in order to promote the perceived occupational safety climate.

For two out of three indices, we also found significant group differences with a medium to large effect. Occupational safety measures initiated by the employer were considered more important by nurses than by physicians. Here it can be assumed that the questioned nurses desire more regulations with regard to occupational safety on the part of the employer. Additionally, in our sample, nurses indicated occupational risks more often than physicians. This result has not been described previously and is surprising, since within their profession physicians have more invasive activities than nurses. Studies also show that physicians, for example, are more affected by needlestick injuries than nurses [74, 75]. Therefore, we cannot explain why nurses in our sample indicated occupational risks more often than physicians.

### Strengths and limitations

In our study, we assessed psychosocial working conditions, leadership, patient safety climate, and occupational safety climate in one standardized questionnaire. The identified results in the different four topics can help to identify where improvements for either professional group or a specific emphasis on certain topics are necessary. Based on the results, we can derive further implications to finally improve working conditions, leadership, patient safety climate, and occupational safety climate in hospitals for nurses and physicians. Our results show for example that high quantitative demands should be reduced and also that adequate staffing may contribute to improved patient safety. In addition, it seems necessary for hospital management to become more visible by actively supporting measures for improved patient and occupational safety climate.

This study also has some limitations. First, the results from the cross-sectional study only refer to one point of time. The survey was conducted at only two university hospitals in Germany, and we had an overall response rate of 39.6%. We excluded units with specific treatment in patient care, such as intensive care and psychiatric units. Therefore, presented results are limited with regard to generalizability, but should at least be applicable to other university hospitals in Germany. Second, the questionnaire covered only self-reports by physicians and nurses. We did not include the perspectives from hospital management. To comprehensively measure patient and occupational safety climate, a combination of different methods, such as survey and observation, should be used. In addition, the perspective from other professional groups and from patients could be valuable to evaluate these four topics and to develop improvements in these areas.

## Conclusions

The WorkSafeMed study combined the assessment of four topics: Psychosocial *working conditions*, *leadership*, *patient safety climate*, and *occupational safety climate* in hospitals. Considering nurses’ and physician’s perceptions of these four perspectives provides an integrative overview of where improvements may be needed in hospitals. There were, in part, great differences in the evaluation of these four topics by the two professional groups included in this study. For example, psychosocial working conditions and patient safety climate were assessed more positively by physicians than by nurses. These results may help to refine how different professional groups are addressed when aiming for improvements that are meaningful based on their most pressing needs.

## Additional file

Additional file 1: Descriptive statistics, results of the student’s t test and effect size comparing answers by study participants of the two university hospitals. (DOCX 38 kb)

## Abbreviations

CBI: Copenhagen Burnout Inventory
COPSOQ: Copenhagen Psychosocial Questionnaire
HSPSC: Hospital Survey on Patient Safety Culture
TLI: Transformational Leadership Inventory
