# Correction to: Healthcare professionals’ perspectives on working conditions, leadership, and safety climate: a cross-sectional study

**DOI:** 10.1186/s12913-019-4838-y

**Published:** 2020-01-22

**Authors:** Anke Wagner, Monika A. Rieger, Tanja Manser, Heidrun Sturm, Juliane Hardt, Peter Martus, Constanze Lessing, Antje Hammer, on behalf of the WorkSafeMed Consortium

**Affiliations:** 1 Institute of Occupational and Social Medicine and Health Services Research, University Hospital of Tübingen, Wilhelmstraße 27, 72074 Tübingen, Germany; 2 University of Applied Sciences and Arts Northwestern Switzerland, FHNW School of Applied Psychology, Riggenbachstrasse 16, 4600 Olten, Switzerland; 3 Institute for Clinical Epidemiology and Applied Biometry, University Hospital of Tübingen, Silcherstraße 5, 72076 Tübingen, Germany; 4 Berlin Institute of Health (BIH), Clinical Research Unit (CRU), Anna-Louisa-Karsch-Str. 2, 10178 Berlin, Germany; 5 Institute of Biometry and Clinical Epidemiology, Charité –Universitätsmedizin Berlin, Charitéplatz 1, 10117 Berlin, Germany; 6 Institute for Patient Safety, University Hospital of Bonn, Sigmund-Freud-Str. 25, 53127 Bonn, Germany; 7 independent consultant, Berlin, Germany

**Correction to: BMC Health Serv Res**

**https://doi.org/10.1186/s12913-018-3862-7**

In the original publication of this article [1], the authors missed that reverse coding was necessary for the item “Do you work separate from your colleagues?” before calculating the scale ‘social relations’. So they corrected the analysis accordingly. The results with the revised scale show that there are no longer any significant differences between nurses and physicians with regard to this scale.

This error (scale social relations) affects the following parts of our manuscript:

**‘Methods’ section:**

Old version: We also adapted one scale from the Copenhagen Burnout Inventory (client-related burnout) to measure patient-related burnout [54]. Before calculating scale scores for each dimension and in accordance with the recommended COPSOQ transformation [52], scales were transformed into scores ranging from 0 (minimum value, “do not agree at all”) to 100 points (maximum value, “fully agree”).

Correction: We also adapted one scale from the Copenhagen Burnout Inventory (client-related burnout) to measure patient-related burnout [54]. Before scale calculation, reverse coding was carried out for one item (“Do you work separate from your colleagues?”). Scale calculation was done in accordance with the recommended COPSOQ transformation [52], scales were transformed into scores ranging from 0 (minimum value, “do not agree at all”) to 100 points (maximum value, “fully agree”).

**‘Result’ section:**

Old version: There were no statistically significant differences between the two professional groups in the four scales predictability, role clarity, feedback, and sense of community.

Correction: There were no statistically significant differences between the two professional groups in the five scales predictability, role clarity, feedback, social relations, and sense of community.

Old version: We identified significant differences with small or medium effects in three scales (social support: d = −.15, role conflicts: d = −.31, and social relations: d = .40).

Correction: We identified significant differences with small or medium effects in two scales (social support: d = −.15, and role conflicts: d = −.31).

Old version: Physicians rated items on the scale social relations more positively (51.5 ± 15.1) than the nurses (45.0 ± 17.0).

Correction: Values for the scale social relations were relatively high for both physicians (54.8 ± 20.7) and nurses (55.5 ± 20.2). There was no significant difference between the two professional groups in rating the scale social relations.

**‘Discussion’ section:**

Old version: Our study found significant differences between the two professional groups in 12 out of 17 scales. Nine scales (influence at work, degree of freedom at work, possibilities for development, meaning of work, workplace commitment, role conflicts, social relations, job satisfaction, and the additional scale patient-related burnout) were significantly more positively assessed by physicians than the nursing staff),

Corrected version: Our study found significant differences between the two professional groups in 11 out of 17 scales. Eight scales (influence at work, degree of freedom at work, possibilities for development, meaning of work, workplace commitment, role conflicts, job satisfaction, and the additional scale patient-related burnout) were significantly more positively assessed by physicians than the nursing staff.

**Revised Table 3**

We corrected the values for the scale “Social relations”. We also detected some minor errors with no consequences and corrected them too (for the following scales or single items: “Emotional demands”, “Teamwork within units”, “My direct supervisor focuses more on patient safety than a year ago”, “Hospital management openly addresses problems concerning patient safety in our hospital”, “Hospital management focuses more on patient safety than a year ago” and “My direct supervisor openly addresses problems concerning occupational safety in our hospital”.

Table 3 with the corrected values is shown below:

**Table 3:** Descriptive statistics, results of the student’s t test and effect size comparing answers by nurses and physicians

Psychosocial working conditions	Interpretation (0=minimum value, 100=maximum value)	Mean (SD) (nurses=564)	Mean (SD) (physicians=380)	(df) t-value^1^	d_Cohen_
Copenhagen Psychosocial Questionnaire (COPSOQ)					
Quantitative demands	high=negative	66.5 (13.5)	71.9 (13.9)	(942) -5.974*	0.40
Emotional demands	high=negative	64.4 (18.3)	64.6 (16.5)	(866) -0.206	0.01
Work-privacy-conflict	high=negative	61.3 (24.4)	68.7 (25.1)	(942) -4.497*	0.30
Influence at work	high=positive	36.3 (17.3)	38.8 (20.8)	(710) -2.006*	0.13
Degree of freedom at work	high=positive	36.0 (15.9)	46.2 (20.0)	(687) -8.373*	0.58
Possibilities for development	high=positive	71.6 (15.7)	79.6 (14.2)	(942) -8.032*	0.53
Meaning of work	high=positive	77.7 (16.6)	82.9 (16.1)	(942) -4.753*	0.32
Workplace commitment	high=positive	48.4 (18.8)	61.3 (19.2)	(942) -10.220*	0.68
Predictability	high=positive	53.3 (16.4)	52.5 (19.3)	(720) 0.710	-0.05
Role clarity	high=positive	73.5 (14.5)	72.5 (16.5)	(740) 1.027	-0.07
Role conflicts	high=negative	50.6 (17.2)	45.1 (18.4)	(942) 4.611*	-0.31
Feedback	high=positive	41.9 (21.0)	41.0 (21.5)	(942) 0.632	-0.04
Social support	high=positive	66.7 (17.0)	64.2 (17.0)	(942) 2.169*	-0.15
Social relations	high=positive	55.5 (20.2)	54.8 (20.7)	(942) 0.512	-0.03
Sense of community	high=positive	77.8 (15.2)	76.7 (15.1)	(942) 1.096	-0.07
Outcome scale – Copenhagen Psychosocial Questionnaire (COPSOQ)					
Job satisfaction	high=positive	67.5 (10.2)	73.4 (12.0)	(942) -8.135*	0.54
Outcome scale – Copenhagen Burnout Inventory (CBI, adapted client-related burnout)					
Patient related burnout	high=negative	36.5 (17.6)	28.0 (16.5)	(942) 7.464*	-0.50
Leadership	Interpretation (0/1=minimum value, 100/5=maximum value)	Mean (SD) (nurses=543)	Mean (SD) (physicians=369)	(df) t-value^1^	d_Cohen_
Transformational Leadership Inventory (TLI short)					
Transformational leadership	5=positive	3.1 (0.8)	3.2 (0.8)	(910) -1.605	0.13
Copenhagen Psychosocial Questionnaire (COPSOQ)					
Quality of leadership	high=positive	53.8 (22.7)	49.2 (22.9)	(910) 3.031*	-0.20
Patient safety climate	Interpretation (1=minimum value, 5=maximum value)	Mean (SD) (nurses=558)	Mean (SD) (physicians=373)	(df) t-value^1^	d_Cohen_
Hospital Survey on Patient Safety Culture (HSPSC-D)					
Staffing	5=positive	2.4 (0.8)	2.8 (0.8)	(929) -7.721*	0.50
Organizational learning	5=positive	3.0 (0.7)	3.1 (0.7)	(762) -1.366	0.14
Communication openness	5=positive	3.7 (0.6)	3.4 (0.7)	(758) 6.010*	-0.47
Feedback & communication about error	5=positive	3.4 (0.8)	3.3 (0.9)	(929) 1.519	-0.12
Nonpunitive response to error	5=positive	3.3 (0.8)	3.5 (0.8)	(929) -3.746*	0.25
Teamwork within units	5=positive	3.3 (0.6)	3.4 (0.6)	(929) -1.326	0.17
Teamwork across units	5=positive	3.0 (0.6)	3.1 (0.7)	(698) -3.316*	0.16
Handoffs & transitions	5=positive	3.2 (0.6)	2.9 (0.7)	(713) 5.702*	-0.47
Supervisor/ manager expectations	5=positive	3.4 (0.7)	3.3 (0.7)	(929) 1.020	-0.14
Management support for patient safety	5=positive	2.6 (0.8)	3.0 (0.8)	(929) -5.797*	0.50
Outcome scales – Hospital Survey on Patient Safety Culture (HSPSC-D)					
Frequency of event reported	5=positive	3.0 (1.1)	2.9 (0.9)	(874) 1.053	-0.10
Overall perceptions of patient safety	5=positive	2.9 (0.7)	3.3 (0.8)	(929) -7.782*	0.54
Patient safety grade	1=positive	2.9 (0.8)	2.6 (0.7)	(929) 7.456*	-0.39
Safety grade in the medication process	1=positive	3.0 (0.8)	2.8 (0.7)	(831) 5.065*	-0.26
Patient safety climate	Interpretation (1=minimum value, 5=maximum value)	Mean (SD) (nurses=543)	Mean (SD) (physicians=369)	(df) t-value^1^	d_Cohen_
TWINS Patient Safety					
Supervisor support for patient safety	5=positive	3.4 (0.8)	3.5 (0.7)	(910) -1.996*	0.13
My direct supervisor openly addresses problems concerning patient safety in our hospital	5=positive	3.3 (0.9)	3.3 (1.0)	(729) -0.865	0.00
My direct supervisor focuses more on patient safety than a year ago	5=positive	2.8 (0.9)	2.8 (1.0)	(735) -0.027	0.00
It is important to my direct supervisor that our hospital pays great attention to patient safety	5=positive	3.4 (0.9)	3.5 (0.9)	(910) -1.509	0.11
Hospital management openly addresses problems concerning patient safety in our hospital	5=positive	2.7 (0.8)	3.0 (0.9)	(910) -4.188*	0.36
Hospital management focuses more on patient safety than a year ago	5=positive	2.7 (0.9)	2.8 (0.9)	(910) -2.758*	0.11
It is important to the Hospital management that our hospital pays great attention to patient safety	5=positive	3.0 (1.0)	3.2 (1.0)	(784) -3.698*	0.20
Do you have an individual influence on how well patient safety is implemented at the workplace	1=positive	3.2 (0.9)	2.9 (1.0)	(910) 4.558*	-0.32
Occupational safety climate	Interpretation (1=minimum value, 5=maximum value)	Mean (SD) (nurses=543)	Mean (SD) (physicians=369)	(df) t-value^1^	d_Cohen_
TWINS Occupational Safety					
Supervisor support for occupational safety	5=positive	3.5 (0.8)	3.4 (0.8)	(910) 1.050	-0.13
My direct supervisor openly addresses problems concerning occupational safety in our hospital	5=positive	3.2 (0.9)	3.2 (0.9)	(910) 0.869	0.00
My direct supervisor focuses more on occupational safety than a year ago	5=positive	2.8 (0.9)	2.7 (0.9)	(910) 0.628	-0.11
It is important to my direct supervisor that our hospital pays great attention to occupational safety	5=positive	3.3 (0.9)	3.2 (1.0)	(910) 2.299*	-0.11
Hospital management openly addresses problems concerning occupational safety in our hospital	5=positive	2.9 (0.9)	3.1 (0.9)	(910) -3.337*	0.22
Hospital management focuses more on occupational safety than a year ago	5=positive	2.7 (0.9)	2.8 (0.9)	(910) -1.936	0.11
It is important to the Hospital management that our hospital pays great attention to occupational safety	5=positive	2.9 (0.9)	3.1 (1.0)	(766) -2.720*	0.21
Do you have an individual influence on how well occupational safety is implemented at the workplace	1=positive	3.3 (0.9)	3.3 (1.0)	(910) 0.893	0.00
Occupational safety climate	Interpretation (1=minimum value, 5=maximum value)	Mean (SD) (nurses=560)	Mean (SD) (physicians=372)	(df) t-value^1^	d_Cohen_
Outcome scales – self constructed indices					
Subjective assessment of specific protective measures (behaviour & regulations) related to infectious diseases	1=positive	1.8 (0.6)	1.8 (0.6)	(930) -1.132	0.00
Subjective assessment of occupational safety measures initiated by the employer, related to own safety	1=positive	1.7 (0.6)	2.0 (0.6)	(930) -8.328*	0.50
Personal perception of the frequency of occupational risks	5=positive	3.2 (0.8)	3.5 (0.7)	(853) -5.608*	0.39

Notes: ^1^*p*-value* ≤.05

**‘Additional file 1’: Revised version**

We corrected the values for the scale “Social relations”. We also discovered another minor error (concerning the scale influence at work) and corrected the value too. The table with the corrected values is shown below:

**Table:** 

Psychosocial working conditions	**Interpretation** **(0=minimum value, 100=maximum value)**	**Mean (SD)** **(hospital 1 = 573)**	**Mean (SD)** **(hospital 2 = 418)**	**(df) t-value**^**1**^	**d**_**Cohen**_
*Copenhagen Psychosocial Questionnaire (COPSOQ)*					
Quantitative demands	high=negative	68.4 (13.7)	68.9 (14,5)	(989) -0.568	0.04
Emotional demands	high=negative	65.1 (17.7)	63.1 (17,8)	(989) 1.742	-0.11
Work-privacy-conflict	high=negative	62.2 (25.5)	66.0 (24,8)	(989) -2.332*	0.15
Influence at work	high=positive	36.1 (19.1)	38.9 (18,5)	(989) -2.295*	0.15
Degree of freedom at work	high=positive	39.8 (18.5)	40.9 (18,4)	(989) -0.926	0.06
Possibilities for development	high=positive	75.2 (16.2)	74.3 (15,5)	(989) 0.896	-0.06
Meaning of work	high=positive	80.6 (16.0)	78.5 (17,7)	(989) 1.918	-0.13
Workplace commitment	high=positive	55.0 (18.8)	51.8 (21,7)	(820) 2.447*	-0.16
Predictability	high=positive	54.7 (17.0)	50.7 (18,5)	(989) 3.452*	-0.23
Role clarity	high=positive	74.3 (15.3)	71.6 (15,9)	(989) 2.746*	-0.17
Role conflicts	high=negative	47.3 (17.3)	49.9 (18,9)	(989) -2.267*	0.15
Feedback	high=positive	40.1 (20.7)	43.5 (22,0)	(866) -2.418*	0.16
Social support	high=positive	66,0 (16.4)	65.4 (17,7)	(858) 0.587	-0.04
Social relations	high=positive	55.4 (20.5)	55.9 (20.8)	(989) -0.401	0.02
Sense of community	high=positive	78.1 (14.8)	76.2 (15,2)	(989) 1.949	-0.13
*Outcome scale – Copenhagen Psychosocial Questionnaire (COPSOQ)*					
Job satisfaction	high=positive	70.4 (11.1)	69.3 (11.7)	(989) 1.475	-0.10
*Outcome scale – Copenhagen Burnout Inventory (CBI, adapted client-related burnout)*					
Patient related burnout	high=negative	33.4 (17.4)	32.1 (18.0)	(989) 1.141	-0.07
Leadership	**Interpretation** **(0/1=minimum value, 100/5=maximum value)**	**Mean (SD)** **(hospital 1 = 544)**	**Mean (SD)** **(hospital 2 = 409)**	**(df) t-value**^**1**^	**d**_**Cohen**_
***Transformational Leadership Inventory (TLI short)***					
Transformational leadership	5=positive	3.2 (0.8)	3.2 (0.8)	(951) 0.191	0.00
***Copenhagen Psychosocial Questionnaire (COPSOQ)***					
Quality of leadership	high=positive	52.7 (22.6)	51.0 (23.4)	(951) 1.095	-0.07
**Patient safety climate**	**Interpretation** **(1=minimum value, 5=maximum value)**	**Mean (SD)** **(hospital 1 = 560)**	**Mean (SD)** **(hospital 2 = 414)**	**(df) t-value**^**1**^	**d**_**Cohen**_
***Hospital Survey on Patient Safety Culture (HSPSC-D)***					
Staffing	5=positive	2.5 (0.8)	2.6 (0.8)	(972) -0.965	0.13
Organizational learning	5=positive	3.1 (0.7)	3.0 (0.7)	(972) 0.758	-0.14
Communication openness	5=positive	3.6 (0.7)	3.5 (0.7)	(972) 2.207*	-0.14
Feedback & communication about error	5=positive	3.4 (0.8)	3.3 (0.9)	(972) 2.315*	-0.12
Nonpunitive response to error	5=positive	3.5 (0.8)	3.2 (0.8)	(843) 4.585*	-0.38
Teamwork within units	5=positive	3.4 (0.6)	3.3 (0.6)	(972) 1.669	-0.17
Teamwork across units	5=positive	3.1 (0.6)	3.0 (0.6)	(972) 1.800	-0.17
Handoffs & transitions	5=positive	3.1 (0.6)	3.0 (0.6)	(972) 2.187*	-0.17
Supervisor/ manager expectations	5=positive	3.3 (0.7)	3.3 (0.7)	(972) -0.273	0.00
Management support for patient safety	5=positive	2.8 (0.9)	2.7 (0.9)	(972) 1.579	-0.11
***Outcome scales – Hospital Survey on Patient Safety Culture (HSPSC-D)***					
Frequency of event reported	5=positive	3.0 (1.0)	3.0 (1.0)	(972) -0.191	0.00
Overall perceptions of patient safety	5=positive	3.0 (0.8)	3.1 (0.8)	(972) -1.262	0.13
Patient safety grade	1=positive	2.8 (0.8)	2.8 (0.7)	(972) 0.405	0.00
Safety grade in the medication process	1=positive	2.8 (0.7)	3.0 (0.8)	(972) -2.730*	0.27
**Patient safety climate**	**Interpretation** **(1=minimum value, 5=maximum value)**	**Mean (SD)** **(hospital 1 = 544)**	**Mean (SD)** **(hospital 2 = 409)**	**(df) t-value**^**1**^	**d**_**Cohen**_
***TWINS Patient Safety (TWINS-PS)***					
Supervisor support for patient safety	5=positive	3.5 (0.8)	3.5 (0.8)	(951) 0.702	0.00
My direct supervisor openly addresses problems concerning patient safety in our hospital	5=positive	3.3 (0.9)	3.3 (0.9)	(951) -0.794	0.00
My direct supervisor focuses more on patient safety than a year ago	5=positive	2.8 (0.9)	2.8 (1.0)	(847) 0.191	0.00
It is important to my direct supervisor that our hospital pays great attention to patient safety	5=positive	3.5 (0.9)	3.5 (0.9)	(951) 0.380	0.00
Hospital management openly addresses problems concerning patient safety in our hospital	5=positive	2.9 (0.8)	2.8 (0.9)	(864) 2.555*	-0.12
Hospital management focuses more on patient safety than a year ago	5=positive	2.7 (0.9)	2.8 (0.9)	(951) -0.382	0.11
It is important to the Hospital management that our hospital pays great attention to patient safety	5=positive	3.2 (0.9)	3.0 (1.0)	(951) 2.344*	-0.21
Do you have an individual influence on how well patient safety is implemented at the workplace	1=positive	3.1 (0.9)	3.0 (1.0)	(951) 1.434	-0.11
**Occupational safety climate**	**Interpretation** **(1=minimum value, 5=maximum value)**	**Mean (SD)** **(hospital 1 = 544)**	**Mean (SD)** **(hospital 2 = 409)**	**(df) t-value**^**1**^	**d**_**Cohen**_
***TWINS Occupational Safety (TWINS-OS)***					
Supervisor support for occupational safety	5=positive	3.5 (0.8)	3.4 (0.8)	(951) 0.736	-0.13
My direct supervisor openly addresses problems concerning occupational safety in our hospital	5=positive	3.3 (0.9)	3.2 (0.9)	(951) 1.683	-0.11
My direct supervisor focuses more on occupational safety than a year ago	5=positive	2.8 (0.9)	2.8 (0.9)	(853) 0.852	0.00
It is important to my direct supervisor that our hospital pays great attention to occupational safety	5=positive	3.3 (0.9)	3.2 (1.0)	(951) 1.252	-0.11
Hospital management openly addresses problems concerning occupational safety in our hospital	5=positive	3.1 (0.9)	2.9 (0.9)	(951) 2.470*	-0.22
Hospital management focuses more on occupational safety than a year ago	5=positive	2.7 (0.9)	2.7 (1.0)	(820) 0.220	0.00
It is important to the Hospital management that our hospital pays great attention to occupational safety	5=positive	3.1 (1.0)	3.0 (1.0)	(951) 1.193	-0.10
Do you have an individual influence on how well occupational safety is implemented at the workplace	1=positive	3.3 (0.9)	3.3 (1.0)	(951) 0.826	0.00
**Occupational safety climate**	**Interpretation** **(1=minimum value, 5=maximum value)**	**Mean (SD)** **(hospital 1 = 560)**	**Mean (SD)** **(hospital 2 = 413)**	**(df) t-value**^**1**^	**d**_**Cohen**_
***Outcome scales – self constructed indices s***					
Subjective assessment of specific protective measures (behaviour & regulations) related to infectious diseases	1=positive	1.8 (0.6)	1.8 (0.6)	(971) 0.396	0.00
Subjective assessment of occupational safety measures initiated by the employer, related to own safety	1=positive	1.8 (0.6)	1.9 (0.6)	(835) -1.632	0.17
Personal perception of the frequency of occupational risks	5=positive	3.4 (0.7)	3.3 (0.8)	(825) 1.870	-0.13

Notes: ^1^*p*-value* ≤.05
