# Supplementary material for: Healthcare professionals’ perspectives on working conditions, leadership, and safety climate: a cross-sectional study
Source: BMC Health Serv Res. 2019 Jan 21;19:53. doi: 10.1186/s12913-018-3862-7 (PMC6341698; doi:10.1186/s12913-018-3862-7)
Supplement: Supplementary file 1 — Descriptive statistics, results of the student’s t test and effect size comparing answers by study participants of the two university hospitals. (DOCX 38 kb) [file 12913_2018_3862_MOESM1_ESM.docx]

**Additional file 1 - Descriptive statistics, results of the student’s t test and effect size comparing answers by study participants of the two university hospitals**

| **Psychosocial working conditions** | **Interpretation**  **(0=minimum value,**  **100=maximum value)** | **Mean (SD)**  **(hospital 1 = 573)** | **Mean (SD)**  **(hospital 2 = 418)** | **(df) t-value^1^** | **d_Cohen_** |
| --- | --- | --- | --- | --- | --- |
| ***Copenhagen Psychosocial Questionnaire (COPSOQ)*** | | | | | |
| Quantitative demands | high=negative | 68,4 (13,7) | 68,9 (14,5) | (989) -0,568 | 0.04 |
| Emotional demands | high=negative | 65,1 (17,7) | 63,1 (17,8) | (989) 1,742 | -0.11 |
| Work-privacy-conflict | high=negative | 62,2 (25,5) | 66,0 (24,8) | (989) -2,332 | 0.15 |
| Influence at work | high=positive | 36,1 (19,1) | 38,9 (18,5) | (989) -0,926 | 0.15 |
| Degree of freedom at work | high=positive | 39,8 (18,5) | 40,9 (18,4) | (989) -0,926 | 0.06 |
| Possibilities for development | high=positive | 75,2 (16,2) | 74,3 (15,5) | (989) 0,896 | -0.06 |
| Meaning of work | high=positive | 80,6 (16,0) | 78,5 (17,7) | (989) 1,918 | -0.13 |
| Workplace commitment | high=positive | 55,0 (18,8) | 51,8 (21,7) | (820) 2,447 | -0.16 |
| Predictability | high=positive | 54,7 (17,0) | 50,7 (18,5) | (989) 3,452 | -0.23 |
| Role clarity | high=positive | 74,3 (15,3) | 71,6 (15,9) | (989) 2,746 | -0.17 |
| Role conflicts | high=negative | 47,3 (17,3) | 49,9 (18,9) | (989) -2,267 | 0.15 |
| Feedback | high=positive | 40,1 (20,7) | 43,5 (22,0) | (866) -2,418 | 0.16 |
| Social support | high=positive | 66,0 (16,4) | 65,4 (17,7) | (858) 0,587 | -0.04 |
| Social relations | high=positive | 46,7 (16,8) | 48,5 (16,2) | (989) -1,728 | 0.11 |
| Sense of community | high=positive | 78,1 (14,8) | 76,2 (15,2) | (989) 1,949 | -0.13 |
| ***Outcome scale – Copenhagen Psychosocial Questionnaire (COPSOQ)*** | | | | |  |
| Job satisfaction | high=positive | 70,4 (11,1) | 69,3 (11,7) | (989) 1,475 | -0.10 |
| ***Outcome scale – Copenhagen Burnout Inventory (CBI, adapted client-related burnout)*** | | | | | |
| Patient related burnout | high=negative | 33,4 (17,4) | 32,1 (18,0) | (989) 1,141 | -0.07 |
| **Leadership** | **Interpretation**  **(0/1=minimum value, 100/5=maximum value)** | **Mean (SD)**  **(hospital 1 = 544)** | **Mean (SD)**  **(hospital 2 = 409)** | **(df) t-value^1^** | **d_Cohen_** |
| ***Transformational Leadership Inventory (TLI short)*** | | | | | |
| Transformational leadership | 5=positive | 3,2 (0,8) | 3,2 (0,8) | (951) 0,191 | 0.00 |
| ***Copenhagen Psychosocial Questionnaire (COPSOQ)*** | | | | | |
| Quality of leadership | high=positive | 52,7 (22,6) | 51,0 (23,4) | (951) 1,095 | -0.07 |
| **Patient safety climate** | **Interpretation**  **(1=minimum value, 5=maximum value)** | **Mean (SD)**  **(hospital 1 = 560)** | **Mean (SD)**  **(hospital 2 = 414)** | **(df) t-value^1^** | **d_Cohen_** |
| ***Hospital Survey on Patient Safety Culture (HSPSC-D)*** | | | | | |
| Staffing | 5=positive | 2,5 (0,8) | 2,6 (0,8) | (972) -0,965 | 0.13 |
| Organizational learning | 5=positive | 3,1 (0,7) | 3,0 (0,7) | (972) 0,758 | -0.14 |
| Communication openness | 5=positive | 3,6 (0,7) | 3,5 (0,7) | (972) 2,207 | -0.14 |
| Feedback & communication about error | 5=positive | 3,4 (0,8) | 3,3 (0,9) | (972) 2,315 | -0.12 |
| Nonpunitive response to error | 5=positive | 3,5 (0,8) | 3,2 (0,8) | (843) 4,585 | -0.38 |
| Teamwork within units | 5=positive | 3,4 (0,6) | 3,3 (0,6) | (972) 1,669 | -0.17 |
| Teamwork across units | 5=positive | 3,1 (0,6) | 3,0 (0,6) | (972) 1,800 | -0.17 |
| Handoffs & transitions | 5=positive | 3,1 (0,6) | 3,0 (0,6) | (972) 2,187 | -0.17 |
| Supervisor/ manager expectations | 5=positive | 3,3 (0,7) | 3,3 (0,7) | (972) -0,273 | 0.00 |
| Management support for patient safety | 5=positive | 2,8 (0,9) | 2,7 (0,9) | (972) 1,579 | -0.11 |
| ***Outcome scales – Hospital Survey on Patient Safety Culture (HSPSC-D)*** | | | | | |
| Frequency of event reported | 5=positive | 3,0 (1,0) | 3,0 (1,0) | (972) -0,191 | 0.00 |
| Overall perceptions of patient safety | 5=positive | 3,0 (0,8) | 3,1 (0,8) | (972) -1,262 | 0.13 |
| Patient safety grade | 1=positive | 2,8 (0,8) | 2,8 (0,7) | (972) 0,405 | 0.00 |
| Safety grade in the medication process | 1=positive | 2,8 (0,7) | 3,0 (0,8) | (972) -2,730 | 0.27 |
| **Patient safety climate** | **Interpretation**  **(1=minimum value, 5=maximum value)** | **Mean (SD)**  **(hospital 1 = 544)** | **Mean (SD)**  **(hospital 2 = 409)** | **(df) t-value^1^** | **d_Cohen_** |
| ***TWINS Patient Safety (TWINS-PS)*** | | | | | |
| Supervisor support for patient safety | 5=positive | 3,5 (0,8) | 3,5 (0,8) | (951) 0,702 | 0.00 |
| My direct supervisor openly addresses problems concerning patient safety in our hospital | 5=positive | 3,3 (0,9) | 3,3 (0,9) | (951) -0,794 | 0.00 |
| My direct supervisor focuses more on patient safety than a year ago | 5=positive | 2,8 (0,9) | 2,8 (1,0) | (847) 0,191 | 0.00 |
| It is important to my direct supervisor that our hospital pays great attention to patient safety | 5=positive | 3,5 (0,9) | 3,5 (0,9) | (951) 0,380 | 0.00 |
| Hospital management openly addresses problems concerning patient safety in our hospital | 5=positive | 2,9 (0,8) | 2,8 (0,9) | (864) 2,555 | -0.12 |
| Hospital management focuses more on patient safety than a year ago | 5=positive | 2,7 (0,9) | 2,8 (0,9) | (951) -0,382 | 0.11 |
| It is important to the Hospital management that our hospital pays great attention to patient safety | 5=positive | 3,2 (0,9) | 3,0 (1,0) | (951) 2,344 | -0.21 |
| Do you have an individual influence on how well patient safety is implemented at the workplace | 1=positive | 3,1 (0,9) | 3,0 (1,0) | (951) 1,434 | -0.11 |
| **Occupational safety climate** | **Interpretation**  **(1=minimum value, 5=maximum value)** | **Mean (SD)**  **(hospital 1 = 544)** | **Mean (SD)**  **(hospital 2 = 409)** | **(df) t-value^1^** | **d_Cohen_** |
| ***TWINS Occupational Safety (TWINS-OS)*** | | | | | |
| Supervisor support for occupational safety | 5=positive | 3,5 (0,8) | 3,4 (0,8) | (951) 0,736 | -0.13 |
| My direct supervisor openly addresses problems concerning occupational safety in our hospital | 5=positive | 3,3 (0,9) | 3,2 (0,9) | (951) 1,683 | -0.11 |
| My direct supervisor focuses more on occupational safety than a year ago | 5=positive | 2,8 (0,9) | 2,8 (0,9) | (853) 0,852 | 0.00 |
| It is important to my direct supervisor that our hospital pays great attention to occupational safety | 5=positive | 3,3 (0,9) | 3,2 (1,0) | (951) 1,252 | -0.11 |
| Hospital management openly addresses problems concerning occupational safety in our hospital | 5=positive | 3,1 (0,9) | 2,9 (0,9) | (951) 2,470 | -0.22 |
| Hospital management focuses more on occupational safety than a year ago | 5=positive | 2,7 (0,9) | 2,7 (1,0) | (820) 0,220 | 0.00 |
| It is important to the Hospital management that our hospital pays great attention to occupational safety | 5=positive | 3,1 (1,0) | 3,0 (1,0) | (951) 1,193 | -0.10 |
| Do you have an individual influence on how well occupational safety is implemented at the workplace | 1=positive | 3,3 (0,9) | 3,3 (1,0) | (951) 0,826 | 0.00 |
| **Occupational safety climate** | **Interpretation**  **(1=minimum value, 5=maximum value)** | **Mean (SD)**  **(hospital 1 = 560)** | **Mean (SD)**  **(hospital 2 = 413)** | **(df) t-value^1^** | **d_Cohen_** |
| ***Outcome scales – self constructed indices s*** | | | | | |
| Subjective assessment of specific protective measures (behaviour & regulations) related to infectious diseases | 1=positive | 1,8 (0,6) | 1,8 (0,6) | (971) 0,396 | 0.00 |
| Subjective assessment of occupational safety measures initiated by the employer, related to own safety | 1=positive | 1,8 (0,6) | 1,9 (0,6) | (835) -1,632 | 0.17 |
| Personal perception of the frequency of occupational risks | 5=positive | 3,4 (0,7) | 3,3 (0,8) | (825) 1,870 | -0.13 |

Notes: ^1^p-value≤.05 is highlighted
